# Manual of Bacteriology

**Published:** 1898-06

**Authors:** 


**Manual of Bacteriology.** By Robert Muir, M.D., and James Ritchie, M.D. Pp. xvi., 519. Edinburgh: Young J. Pentland. 1897.

Of the now numerous manuals dealing with the pathological side of bacteriology, this is one of the best. Without overloading the text with unnecessary details, trifling variations of manipulation, or processes of merely historical interest, the authors have succeeded in presenting a remarkably clear and well ordered account of the most approved methods of handling micro-organisms, followed by a series of chapters describing in detail those which are associated with various diseases. The information given is consistently accurate and up to date, and is illustrated by excellent reproductions of photographs.

An appendix containing equally satisfactory though brief accounts of malarial fever and dysentery brings to a close a work that may be strongly recommended to those who are entering upon the study of micro-organisms in relation to disease.

